# Do apelin and ghrelin play a role in multiple sclerosis? The analysis of patients treated with immunomodulatory therapies

**DOI:** 10.3389/fimmu.2026.1701657

**Published:** 2026-04-28

**Authors:** Bożena Adamczyk, Natalia Morawiec, Michał Rakoca, Agata Sowa, Ksawier Sawa, Monika Adamczyk-Sowa

**Affiliations:** 1 Department of Neurology, Faculty of Medical Sciences in Zabrze, Medical University of Silesia in Katowice, Zabrze, Poland; 2 Department of Neurology, 10th Military Research Hospital and Polyclinic, Bydgoszcz, Poland

**Keywords:** apelin, fingolimod, ghrelin, multiple sclerosis, natalizumab

## Abstract

**Introduction:**

Multiple sclerosis (MS) is an autoimmune disorder with a complex and multifactorial etiology. Increasing evidence suggests that peptide hormones may significantly influence the pathogenesis of MS by affecting inflammatory processes and metabolic dysregulation. The aim of the study was to evaluate the levels of apelin and ghrelin, which are two peptide hormones, in patients with relapsing-remitting MS (RRMS) receiving natalizumab and fingolimod compared to healthy controls.

**Methods:**

We enrolled forty-nine patients with RRMS and thirty-eight healthy individuals. The participants were divided into three groups i.e., natalizumab-treated patients, fingolimod-treated patients, and healthy controls. We assessed the serum levels of apelin and ghrelin. Statistical analysis was performed using STATISTICA 12. In both groups, correlations between the duration of the disease and treatment, BMI and hormone concentrations were evaluated.

**Results:**

The natalizumab-treated group showed significantly higher levels of apelin (p=0.0063), while the fingolimod-treated group showed significantly higher ghrelin levels compared to the controls (p=0.0035). *Post-hoc* analysis revealed that all women treated with natalizumab and fingolimod had higher apelin levels than healthy women (p=0.025). In the group of women with MS, ghrelin levels increased with disease duration (p=0.045, R = 0.357). Additionally, apelin levels correlated positively with BMI in the fingolimod-treated group (p=0.001, R = 0.694).

**Conclusion:**

The study demonstrates differences in serum apelin and ghrelin levels between RRMS patients treated with natalizumab or fingolimod and healthy controls, suggesting a potential association between immunomodulatory treatment, metabolic factors, and peptide hormone regulation in multiple sclerosis.

## 1 Introduction

Multiple sclerosis (MS) is a chronic disease characterized by inflammation and demyelination of the central nervous system (CNS). The disease leads to damage of the myelin sheath and subsequent nerve conduction impairment. In the initial phases of MS, the inflammatory process is predominant. As the disease progresses, it is typically associated with neurodegeneration (1). The activation of T-lymphocytes is a significant factor in the pathogenesis of MS. This may be induced by infections such as the Epstein-Barr virus (EBV) or by exposure to myelin antigens (2). Once activated, T-cells migrate to the CNS, where they can differentiate into various subsets, including Th1, Th2, and Th17 cells. These cells modulate the inflammatory response by secreting proinflammatory and anti-inflammatory cytokines (3, 4).

In addition to immunological mechanisms, oxidative stress and metabolic factors are also involved in the pathogenesis of MS. Obesity is identified as a potential risk factor for disease progression (5, 6). Studies have indicated a higher prevalence of overweight and obesity in patients with MS, although the findings are inconclusive (7, 8). Adipocytokines are bioactive substances secreted by adipose tissue. They regulate energy homeostasis and immune processes, which are crucial for the interaction between metabolism and inflammation. Key adipocytokines include visfatin and resistin, which are involved in the inflammatory response, as well as adiponectin and apelin, which influence glucose and lipid metabolism. Changes in adipocytokine levels may affect the development of MS, thus influencing the function of the immune system and neurodegenerative processes (7, 8).

Relapsing–remitting multiple sclerosis (RRMS) was selected for this study because it represents the most prevalent clinical subtype of MS, typically affecting approximately 85–90% of patients at diagnosis and characterized by episodic inflammatory activity in the central nervous system (CNS) with periods of relapse and remission. RRMS is marked by focal inflammatory demyelination and disruption of the blood–brain barrier, which underlies immune cell infiltration into the CNS and contributes to clinical exacerbations (9).

Focusing on RRMS helps reduce clinical heterogeneity compared with progressive forms of MS, where neurodegenerative mechanisms play a greater role than acute inflammation. This makes RRMS a particularly suitable model in which to investigate how immunomodulatory therapies and metabolic factors may be associated with changes in circulating peptide hormones in relation to disease activity (10).

The aim of our study was to compare the serum levels of selected peptide hormones between the patients with RRMS treated with natalizumab (NT) and fingolimod (FG) and the healthy controls. Furthermore, we assessed the levels of apelin and ghrelin depending on the gender, age, disease duration, expanded disability status scale (EDSS), relapse rate, lesions in brain magnetic resonance imaging (MRI), and the body mass index (BMI).

### 1.1 Apelin

Apelin is a G-protein-coupled transmembrane protein that interacts with the angiotensin II receptor. It plays a significant role in regulating water and electrolyte balance, as well as various signaling pathways in the CNS and the immune response. Apelin is produced by several tissues, including the brain, adipose tissue, gastrointestinal tract, cardiovascular system, lungs, liver, spleen, kidneys, mammary gland, and placenta. While apelin receptor expression in the CNS is found only in moderate amounts, particularly high levels of apelin occur in lungs and kidneys (11). Signoriello et al. indicated that apelin and its receptor were involved in various diseases, including neurological disorders, hypertension, metabolic diseases, respiratory diseases, gastrointestinal diseases, liver disorders, kidney diseases, and cancer. In oncology, apelin acts as a stimulator of tumor growth, and increased levels of apelin serve as an adverse prognostic factor (12). As apelin is produced in the CNS, its relevance in neurodegenerative diseases such as Alzheimer’s, Parkinson’s, and Huntington’s diseases is currently under investigation (13).

The role of apelin in MS remains unclear. The activation of microglia and macrophages is a critical factor in the pathogenesis of MS. Park et al. found a significant reduction in apelin levels in the brains of MS patients and animal models of experimental autoimmune encephalomyelitis (EAE). The study findings suggested its potential involvement in the disease mechanism (14). Apelin shows neuroprotective effects by influencing the function of microglia and macrophages, reducing the expression of inflammatory markers, and promoting remyelination processes. Furthermore, the use of a stable analog of apelin-13 in EAE models has been shown to improve clinical outcomes and reduce the extent of demyelination, which indicated that apelin could be a potential therapeutic target in MS (15).

### 1.2 Ghrelin

Ghrelin is a 28-amino-acid acylated peptide that plays a significant role in appetite regulation. The activation of this hormone is mediated by the ghrelin O-acyltransferase, which facilitates the attachment of an acyl side chain (16). Ghrelin is primarily produced by type A enteroendocrine cells, with limited production in the gastric body, jejunum, duodenum, lungs, genitourinary organs, and pituitary gland. Ghrelin is responsible for the central and peripheral regulation of appetite. It exerts its central effects by acting on specific receptors in the hypothalamus, leading to an increased urge to eat, particularly during periods of starvation (17). The acyl side chain is vital for ghrelin’s orexigenic action, which stimulates food intake and influences gastric emptying (16). Furthermore, ghrelin interacts with the hypothalamus and brainstem nuclei, which are part of the sympathetic nervous system, to enhance food intake and promote carbohydrate consumption as a primary energy source while sparing adipose tissue (16).

Ghrelin may also be involved in the development of obesity. Studies have found that obese individuals tend to have higher post-prandial levels of ghrelin compared to those with a normal BMI. Excessive production of ghrelin has been observed in obese individuals, regardless of their meal intake (18). Furthermore, the administration of ghrelin can increase appetite, improve the nutritional status, and even lower metabolic rates in patients with end-stage cancer (18).

As regards MS, ghrelin might be a factor influencing the disease course. Correale et al. suggested that higher serum ghrelin levels could correlate with neurofilament light (NfL) levels (a marker of axonal damage). This could be significant for assessing disease activity and disability progression. Moreover, together with other adipokines, ghrelin may influence the pathomechanism of MS, affecting neuroinflammatory processes and potentially influencing cognitive function in patients (19).

Beyond its metabolic actions, ghrelin has increasingly been recognized for its immunomodulatory and neuroprotective properties. Experimental studies have demonstrated that ghrelin can attenuate neuroinflammation by regulating microglial activation, suppressing the production of pro-inflammatory cytokines, and promoting neuronal survival in models of central nervous system injury and autoimmune encephalomyelitis (20, 21). Moreover, ghrelin signaling has been shown to protect neurons against oxidative stress and apoptosis, suggesting potential relevance for neurodegenerative mechanisms. Recent genetic and molecular studies further highlight links between metabolic peptides, including ghrelin, and inflammatory signaling pathways involved in MS and related disorders, reinforcing the concept that metabolic regulation and immune function are closely interconnected in neuroinflammatory disease (22). Collectively, these findings provide a strong biological rationale for investigating ghrelin in the context of MS and its modulation by immunomodulatory therapies.

Many studies have examined the role of various substances produced by adipose tissue and the gastrointestinal tract in the development of demyelinating diseases. Our study investigating the effects of natalizumab (NT) and fingolimod (FG) on apelin and ghrelin levels is a novel approach. Understanding their function in the context of MS may provide new therapeutic opportunities.

## 2 Materials and methods

We enrolled 87 participants in the prospective study. The group comprised 49 patients (30 on FG and 19 on NT) diagnosed with RRMS and 38 healthy volunteers. The participants met the following inclusion criteria: a diagnosis of RRMS based on the 2017 McDonald criteria (for patients), treatment with natalizumab or fingolimod, age ≥18 years, written informed consent, and no relapse in the previous six months. Exclusion criteria included lack of consent, coexistence of other autoimmune diseases, use of other drugs or dietary supplements, and current or past smoking. Consequently, all study participants, including patients and healthy controls, were non-smokers. All blood samples were collected under fasting conditions. In patients with RRMS, blood was additionally drawn immediately before administration of the next scheduled dose of natalizumab or fingolimod. All study participants, including healthy controls, had body mass index (BMI) values within the normal range (18.5–24.9 kg/m²).

Blood sampling and pre-analytical procedures. All participants (RRMS patients and healthy controls) were non-smokers, and all blood samples were collected after an overnight fast. In RRMS patients, blood was additionally drawn immediately before administration of the next scheduled dose of natalizumab or fingolimod. Venous blood was collected into standard serum tubes, allowed to clot, and centrifuged; serum was aliquoted and stored at −80 °C until analysis. To minimize pre-analytical variability, samples were thawed only once and repeated freeze–thaw cycles were avoided.

Hormone measurements. Serum apelin and ghrelin concentrations were determined using commercially available ELISA kits (Bio-Techne/Novus Biologicals): Human Apelin ELISA Kit (Colorimetric), catalog # NBP2-68234 (analytical sensitivity 37.5 pg/mL, assay range 62.5–4000 pg/mL, intra-assay CV <5.07%, inter-assay CV <4.93%) and Human Ghrelin ELISA Kit (Colorimetric), catalog # NBP2-66709 (analytical sensitivity 9.38 pg/mL, assay range 15.63–1000 pg/mL, intra-assay CV <5.78%, inter-assay CV <5.08%). Assays were performed according to the manufacturers’ instructions (23).

Microsoft Excel was used to prepare the database. Statistical analyses were conducted using Statistica 12. Descriptive statistics were presented as the arithmetic mean and standard deviation, while qualitative variables were summarized as percentages. To assess the homogeneity of continuous variables across groups, the parametric ANOVA test was used for variables that followed a normal distribution. The non-parametric Kruskal-Wallis ANOVA was applied to variables that did not have a normal distribution. If significant differences were identified, a *post-hoc* analysis was performed using the Tukey test adjusted with Bonferroni correction. The Student’s t-test and the non-parametric Mann-Whitney U test were used to compare the two groups. Correlations among selected variables were assessed using Pearson’s correlation coefficient for normally distributed variables and Spearman’s rank correlation coefficient for non-normally distributed variables. The significance level was set at p<0.05. Statistical error control. All statistical tests were two-sided and a significance level of p<0.05 was considered statistically significant. When global group differences were detected using ANOVA or Kruskal–Wallis tests, *post-hoc* pairwise comparisons were performed with Bonferroni adjustment to control for multiple testing and to limit the risk of type I error. The normality of continuous variables was assessed using the Shapiro–Wilk test. Variables with a normal distribution were analyzed using parametric tests, whereas non-normally distributed variables were analyzed using non-parametric tests.

Exploratory correlation analyses were performed within treatment groups to assess associations between apelin and ghrelin levels and clinical (EDSS, ARR) and MRI measures of disease activity.

The study was conducted in accordance with the principles of the Declaration of Helsinki. The study protocol was approved by the Bioethics Committee of the Silesian Medical University in Katowice (KNW/0022/KB1/37/16).

## 3 Results

Women were predominant in the study group (65.31%). The mean age of the RRMS patients was 36.11 years (**Table 1**).

**Table 1:** General characteristics of the RRMS patients treated with fingolimod or natalizumab and healthy controls.

Variable	Fingolimod (FG)	Natalizumab (NT)	Healthy controls	p
N	30	19	38	—
Age (years)	36.9 ± 11.6	34.8 ± 10.5	39.94 ± 12.03	0.14
Female sex (%)	66.7	63.2	76.3	0.23
BMI (kg/m²)	23.47 ± 2.41	23.93 ± 3.82	23.00 ± 2.32	0.61

RRMS, relapsing-remitting multiple sclerosis.

In the groups treated with FG and NT, disease activity measured by EDSS, annual relapse rate (ARR) and the mean number of Gd(+) lesions and T2-weighted lesions on brain MRI did not differ significantly. The mean disease duration in both groups was 7.46 years and 5.52 years, respectively. The subjects had normal BMI values (**Table 2**).

**Table 2:** General characteristics of the RRMS group with disease duration, BMI, EDSS, ARR, mean number of Gd+ and T2-weighted lesions on brain MRI.

Variable	FG	NT	p
Disease duration (years)	7.46 ± 4.34	5.52 ± 3.23	0.10
BMI (kg/m^2^)	23.47 ± 2.41	23.93 ± 3.82	0.61
EDSS (points)	3.28 ± 1.03	3.11 ± 1.03	0.92
ARR (N)	0.34 ± 0.61	0.32 ± 0.53	0.21
Mean number of Gd(+) lesions on brain MRI (N)	0.11 ± 0.42	0.17 ± 0.71	0.49
Mean number of T2-weighted lesions on brain MRI (N)	19.30 ± 1.65	18.52 ± 2.27	0.32

FG, fingolimod-treated patients, NT, natalizumab-treated patients, EDSS, Expanded Disability Status Scale, ARR, annual relapse rate, BMI, body mass index, MRI, magnetic resonance imaging.

### 3.1 The comparison of the selected peptide hormones in the serum in patients treated with fingolimod and natalizumab compared to the controls

Patients treated with natalizumab exhibited significantly higher serum apelin concentrations compared to fingolimod-treated patients and healthy controls. In contrast, ghrelin concentrations were significantly higher in the FG group than in the controls (**Table 3**). A significant difference was found in apelin and ghrelin concentrations in the study groups. A *post-hoc* analysis was conducted for significant group differences in apelin (**Table 4**) and ghrelin (**Table 5**).

**Table 3:** The comparison of apelin and ghrelin serum concentrations in patients treated with fingolimod and natalizumab compared to the controls.

Variable	NT	FG	Control	p
N	19	30	38	
Apelin (pg/ml)	4559.82 ± 5710.47	1786.46 ± 1576.87	1045.67 ± 1377.83	0.006
Ghrelin (pg/ml)	57.50 ± 18.34	67.17 ± 15.13	56.56 ± 15.01	0.003

FG, fingolimod-treated patients, NT, natalizumab-treated patients.

**Table 4:** *Post-hoc* analysis for apelin in the fingolimod and natalizumab groups and controls.

Group	FG	NT	Control
FG	X	p=0.025	NS
NT	p=0.025	X	p=0.001
Control	NS	p=0.001	x

FG, fingolimod-treated patients, NT, natalizumab-treated patients, NS - no statistical significance; statistical significance at p<0.05.

**Table 5:** *Post-hoc* analysis for ghrelin in the fingolimod and natalizumab groups and controls.

Group	FG	NT	Control
FG	X	NS	p=0.017
NT	NS	X	NS
Control	p=0.017	NS	x

FG, fingolimod-treated patients, NT, natalizumab-treated patients, NS - no statistical significance; statistical significance at p<0.05.

### 3.2 The comparison of the selected peptide hormones in the blood serum in the groups depending on gender

**Figure 1:**
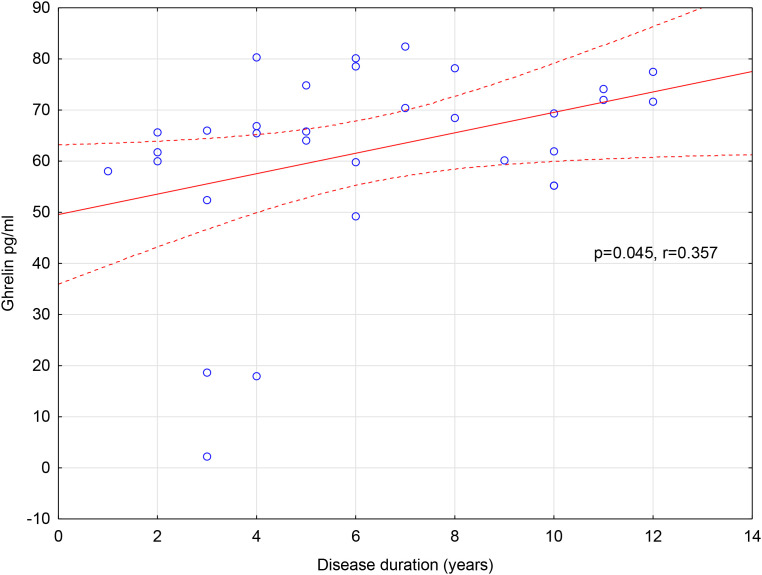
The correlation between disease duration and the level of ghrelin in the group of women with RRMS. RRMS – patients with relapsing-remitting multiple sclerosis treated with natalizumab and fingolimod, statistical significance at p<0.05.

*Post-hoc* analysis showed that women with RRMS had higher apelin levels than healthy women (**Tables 6**, **7**). In addition, ghrelin levels increased with disease duration in women with RRMS (**Figure 1**).

**Table 6:** The comparison of serum concentrations of selected peptide hormones in the RRMS group and controls depending on gender.

Variable	Women with RRMS	Men with RRMS	Women – control group	Men – control group	p
N	32	17	29	9	
Apelin (pg/ml)	2327.94 ± 2218.58	1937.99 ± 1768.25	703.26 ± 553.93	1343.78 ± 1291.42	0.025
Ghrelin (pg/ml)	61.99 ± 18.29	66.05 ± 14.13	56.28 ± 14.90	57.47 ± 16.23	0.071

RRMS, patients with relapsing-remitting multiple sclerosis treated with natalizumab and fingolimod, statistical significance at p<0.05.

**Table 7:** *Post-hoc* analysis for apelin in the groups of women and men with RRMS and the controls.

Group comparison	Women RRMS	Men RRMS	Women – control group	Men – control group
Women with RRMS	NS	NS	p=0.021	NS
Men with RRMS	NS	NS	NS	NS
Women – control group	p=0.021	NS	NS	NS
Men – control group	NS	NS	NS	NS

RRMS – patients with relapsing-remitting multiple sclerosis treated with natalizumab and fingolimod, statistical significance at p<0.05.

### 3.3 The comparison of apelin and ghrelin concentrations in the groups depending on the duration of immunomodulatory treatment

Apelin and ghrelin levels did not differ significantly depending on the duration of immunomodulatory treatment (**Table 8**).

**Table 8:** The comparison of apelin and ghrelin concentrations in the fingolimod-treated group depending on the duration of immunomodulatory treatment.

Treatment duration (months)	<18	>18	p
Apelin (pg/ml)	2145.54 ± 1805.61	2190.38 ± 2046.12	0.909
Ghrelin (pg/ml)	62.95 ± 21.74	63.60 ± 13.40	0.926

### 3.4 The comparison of the concentrations of apelin and ghrelin depending on the BMI

**Figure 2:**
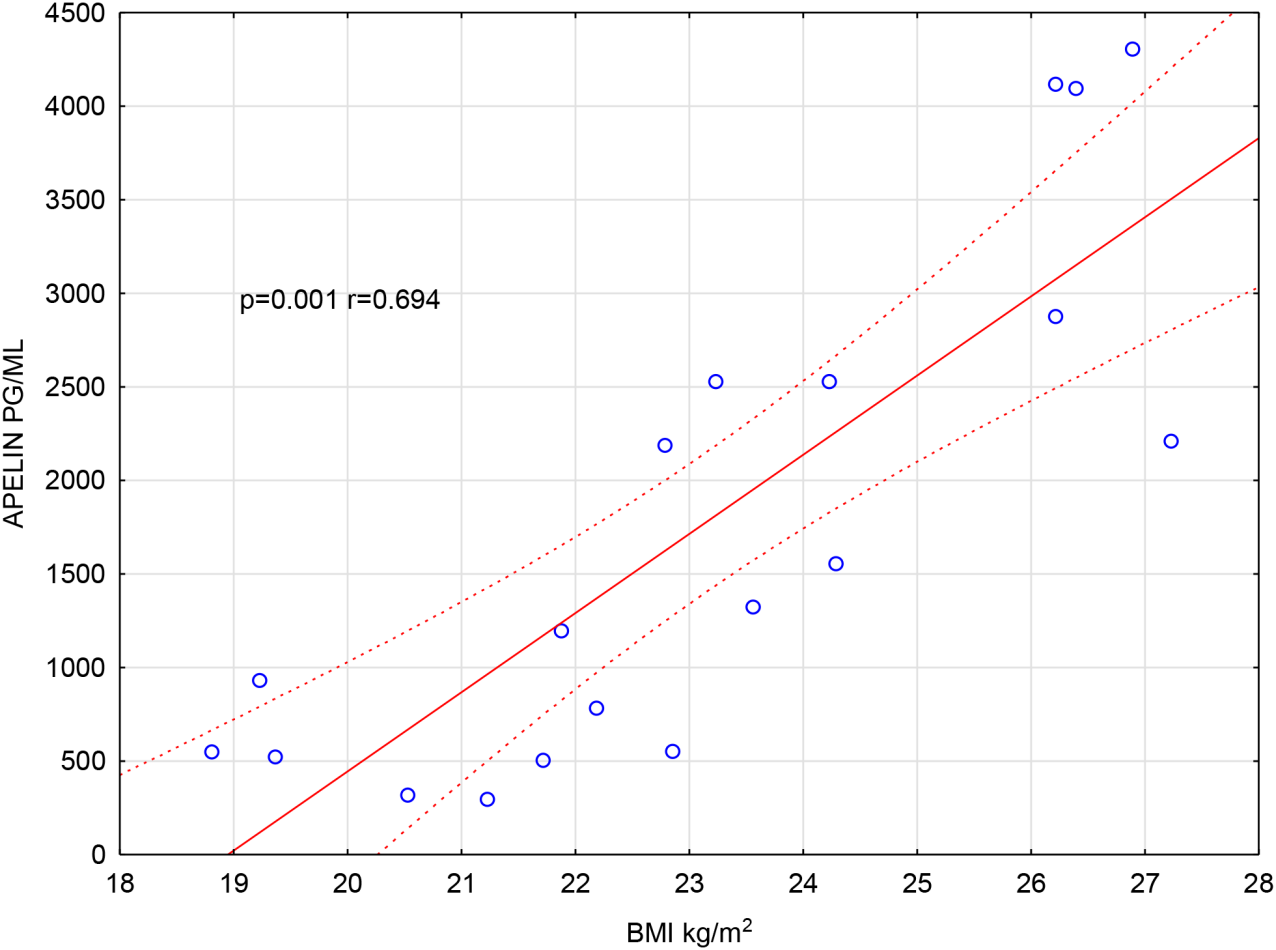
The correlation between apelin levels and BMI in the FG-treated group.

Apelin concentrations correlated positively with BMI in the FG-treated group (**Figure 2**). No significant correlations were found in the NT group.

Within the natalizumab- and fingolimod-treated groups, no significant correlations were found between serum apelin or ghrelin levels and clinical or radiological measures of disease activity, including EDSS score, annual relapse rate (ARR), or the number of Gd-enhancing and T2-weighted MRI lesions (all p > 0.05).

## 4 Discussion

Multiple sclerosis is characterized by chronic inflammation and neurodegeneration of the CNS. In particular, damage to the myelin sheath around axons is caused by the interaction of genetic and environmental factors. Metabolic disorders are also suspected to be involved (24, 25). Overweight and obesity, as the factors that influence the initiation of various diseases, including those of autoimmune origin, have been the subject of many papers. Versini et al. presented data on the relationship between obesity, adipocytokines, and various immune diseases. They showed that the risk of developing MS in obese individuals was significantly higher (26). In particular, obesity during adolescence could become a potentially modifiable risk factor for the development of MS (27). Furthermore, obesity can worsen the course of autoimmune diseases. Adipose tissue is considered an endocrine organ. Adipose tissue cells secrete many pro-inflammatory cytokines. There are many biochemical links between chronic inflammation, which is present in obesity, and the development of autoimmune diseases. In addition, CNS immune cells communicate with systemic immune cells using a variety of transmitters, including adipocytokines and neuropeptides (28).

To date, peptide hormone levels have not been studied in patients treated with NT or FG. Different protocols with NT and FG are highly effective immunomodulatory treatments for MS patients. In the present observational study, higher serum apelin levels were observed in RRMS patients treated with natalizumab compared to other study groups. Given the cross-sectional design, these findings should be interpreted as group-level associations rather than direct treatment effects. It may be related to the direct effect of NT associated with blocking the binding of integrin α4β1 to its receptor and osteopontin ligands (29), as well as to the pathomechanism of MS.

Apelin shows neuroprotective effects (30). The peptide exerts various effects, being involved in fluid homeostasis, immune response, regulation of cardiovascular function, and cell proliferation (31). Some studies have demonstrated that apelin reduces oxidative stress in tissues (32). The most active form of apelin is apelin-13 (30), which may be one of the biomarkers of oxidative stress (33). Elevated oxidative stress parameters are noted in MS patients. Therefore, determination of apelin in this group of patients may be important. Studies on apelin are inconclusive. Alpua et al. showed significantly higher levels of apelin-13 in a small group of patients with MS (42 subjects) compared to healthy individuals (30). Another study reported the opposite results and showed significantly lower levels of apelin in women with MS (34). Considering how many functions apelin has, studies on a large group of patients are warranted to clarify its role in MS.

Birmpli et al. showed lower levels of apelin in the CNS in patients with MS and animal models (EAE). In the EAE model, apelin levels decreased even before the onset of clinical symptoms, which suggests its potential role as a biomarker of the early stages of the disease (15). Apelin-13 influenced the activity of microglia and macrophages, reducing the expression of inflammatory markers (e.g. STAT1) and increasing their phagocytic capacity, which may promote regenerative processes in the CNS. Fluorinated apelin-13 effectively penetrates the CNS when the blood-brain barrier is impaired (e.g., in EAE), which makes it a potential candidate for future neuroprotective therapies in MS. The observed differences in apelin concentrations among treatment groups represent an interesting and novel finding; however, they do not allow conclusions regarding the direct mechanisms of action of natalizumab. Longitudinal studies are required to determine whether these associations reflect treatment-related effects or disease-related factors (15).

Further analysis of the study group showed higher ghrelin levels in patients treated with FG compared to healthy controls. Interpreting the results may be difficult because fasting ghrelin levels were not assessed in all patients. Ghrelin may show neuroprotective properties (35). The synergistic effects of ghrelin and FG may be increased, showing a beneficial therapeutic effect. Based on the above, FG treatment may affect the concentration of some adipocytokines. However, this relationship is less pronounced than in the group treated with NT.

Ghrelin is mainly produced by the stomach. Furthermore, it can exert immunomodulatory effects on the CNS. It is mainly produced outside the CNS. However, specific receptors for this protein are found in nervous tissue (6). Some papers have indicated that ghrelin has anti-inflammatory and neuroprotective properties (35). Both patients with RRMS and SPMS had fasting serum ghrelin levels significantly higher than the controls, while no significant difference was reported between ghrelin levels in patients with different phenotypes of MS. The limitation of their study was small sample size (40 vs. 20) (35). Ghrelin was determined only in serum. Similar assays should also be performed in the cerebrospinal fluid (CSF) in the future. Their study found that ghrelin levels in the CSF were higher in patients with MS (p <0.02) compared to the controls. In addition, CSF concentrations correlated with serum concentrations, which was not noted in the control group. The correlation between serum and CSF concentrations in MS suggests a differential regulation of blood-to-brain transport mechanisms for ghrelin in MS, as reported by the authors (36).

Rats with EAE showed significant improvement after the administration of ghrelin. This improvement was reflected by a decrease in the expression of pro-inflammatory cytokines. The decreased expression of proteins involved in the apoptosis pathway was also reported. The anti-inflammatory effect of ghrelin was associated with inhibition of nuclear factor κB activation. However, rats in the control group did not show statistically significant changes in histologic examinations, pro-inflammatory cytokine production, or molecules involved in the apoptosis signaling pathway. Liu et al. suggested that ghrelin could become a new drug in MS (37), which should be treated with caution.

Another study showed that the administration of ghrelin to rats with EAE resulted in an improvement in the course of the disease. This therapeutic effect was exerted by reducing the autoimmune response. Ghrelin decreased the activation of Th1 and Th17 cells in the nervous system, reduced the number of different inflammatory mediators, and induced regulatory T cells. In conclusion, the authors were of the opinion that ghrelin could be part of a novel therapeutic approach to treat MS (38).

According to some hypotheses, ghrelin concentrations may modify the course of the disease. Studies have not confirmed that genetic variation in the leptin/ghrelin system contributes to the pathogenesis of MS (39).

To date, no studies have comprehensively evaluated the effect of FG treatment on adipocytokine levels. FG may show neuroprotective effects (40) but the possible involvement of ghrelin in this mechanism has not been investigated.

In our study, patients with MS had normal body mass (BMI 23.47 ± 2.41kg/m^2^ for the FG group and 23.93 ± 3.82kg/m^2^ for the NT group). According to other studies, the BMI value was significantly lower in patients with MS during the course of the disease compared to healthy individuals (41). It seems that the value of BMI in women with MS may be of greater importance. Akbay et al. showed a correlation between BMI and higher EDSS scores in women but lower EDSS scores among men. In their study, no correlation was found between BMI and EDSS in the entire cohort (42).

In the FG-treated group, an increase in anti-inflammatory apelin was found when BMI increased. Immunomodulatory treatment can increase apelin levels in women with RRMS. Furthermore, ghrelin levels also increased with treatment duration in this group. We also showed higher concentrations of apelin in the group of women. Female patients undergoing immunomodulatory treatment may be more susceptible to changes in the concentrations of selected peptide hormones than healthy women.

An interesting issue is also the neuroprotective effect of ghrelin in relation to gender (35). Studies have confirmed that MS is more prevalent among women and hormones may play a significant role in the pathogenesis of the disorder (43). Female hormones and genetic predisposition may be involved (44). However, some studies do not support this relationship. A study that evaluated serum concentrations of leptin, ghrelin, testosterone, the ghrelin/leptin ratio, and the testosterone/leptin ratio (P <0.05) showed no differences between women and men with RRMS compared to controls. The authors of the study did not confirm a significant association between the selected peptide hormones and sex hormones in patients with MS and healthy individuals (43).

Understanding the impact of adipocytokines on the course of MS may result in the development of innovative therapies with their many applications in the future. Attempts have already been made to administer ghrelin in autoimmune encephalomyelitis. The initial results seem very encouraging (37, 38). Interestingly, both ghrelin and FG may show potential neuroprotective properties (35, 40).

Our study found no significant correlations for the selected markers of clinical and radiological disease activity (ARR, EDSS, MRI) or the peptide hormones. These analyses were performed within treatment groups and should be regarded as exploratory. Given the limited sample size and the number of clinical and radiological variables, multivariable regression models were not applied to avoid overfitting and unstable estimates. Therefore, the lack of significant associations should not be interpreted as evidence of absence of a biological relationship.

Positive correlations were observed for BMI and apelin in patients treated with FG. Because multiple exploratory comparisons were performed, the results should be interpreted with caution and considered hypothesis-generating despite the use of Bonferroni-adjusted *post-hoc* tests.

Obesity at a young age is a risk factor for MS. However, the value of the BMI does not clearly influence the increase in the risk. Total body fat appears to be more important than the BMI (45). Another important aspect is the relationship between adipocytokine concentrations and gender. In our study, women with MS had higher levels of apelin and an increase in ghrelin levels. The influence of endocrine metabolism is significant, which warrants further investigation. The evaluation of the selected serum peptide hormones in patients treated with NT and FG is a novel approach providing new information to the etiopathogenesis of MS.

Importantly, due to the observational and cross-sectional nature of the study, causality cannot be inferred, and all observed relationships should be interpreted as associations between clinical characteristics, treatment groups, and peptide hormone levels.

## 5 Conclusions

Our findings indicate that RRMS patients treated with natalizumab and fingolimod differ in serum apelin and ghrelin levels compared to healthy controls. These results suggest an association between immunomodulatory treatment status, metabolic factors, and peptide hormone concentrations in multiple sclerosis; however, causal relationships cannot be established based on the present study design.

## Data Availability

The original contributions presented in the study are included in the article/Supplementary Material. Further inquiries can be directed to the corresponding author.
